# The effectiveness of treadmill and swimming exercise in an animal model of osteoarthritis

**DOI:** 10.3389/fphys.2023.1101159

**Published:** 2023-02-21

**Authors:** Leandro Almeida da Silva, Anand Thirupathi, Mateus Cardoso Colares, Daniela Pacheco dos Santos Haupenthal, Ligia Milanez Venturini, Maria Eduarda Anastácio Borges Corrêa, Gustavo de Bem Silveira, Alessandro Haupenthal, Fernando Russo Costa do Bomfim, Thiago Antônio Moretti de Andrade, Yaodong Gu, Paulo Cesar Lock Silveira

**Affiliations:** ^1^ Faculty of Sports Science, Ningbo University, Ningbo, China; ^2^ Laboratory of Experimental Phisiopatology, Program of Postgraduate in Health Sciences, Universidade do Extremo Sul Catarinense, Criciúma, Santa Catarina, Brazil; ^3^ Programa de Pós Graduação em Ciências da Reabilitação, Universidade Federal de Santa Catarina, Araranguá, SC, Brazil; ^4^ Graduate Program of Biomedical Science, Herminio Ometto Foundation, Araras, SP, Brazil

**Keywords:** osteoarthritis, physical exercise, inflammation, oxidative stress, histology

## Abstract

**Introduction:** Osteoarthritis (OA) is considered an inflammatory and degenerative joint disease, characterized by loss of hyaline joint cartilage and adjacent bone remodeling with the formation of osteophytes, accompanied by various degrees of functional limitation and reduction in the quality of life of individuals. The objective of this work was to investigate the effects of treatment with physical exercise on the treadmill and swimming in an animal model of osteoarthritis.

**Methods:** Forty-eight male Wistar rats were divided (n=12 per group): Sham (S); Osteoarthritis (OA); Osteoarthritis + Treadmill (OA + T); Osteoarthritis + Swimming (OA + S). The mechanical model of OA was induced by median meniscectomy. Thirty days later, the animals started the physical exercise protocols. Both protocols were performed at moderate intensity. Forty-eight hours after the end of the exercise protocols, all animals were anesthetized and euthanized for histological, molecular, and biochemical parameters analysis.

**Results:** Physical exercise performed on a treadmill was more effective in attenuating the action of pro-inflammatory cytokines (IFN-γ, TNF-α, IL1-β, and IL6) and positively regulating anti-inflammatories such as IL4, IL10, and TGF-β in relation to other groups.

**Discussion:** In addition to maintaining a more balanced oxi-reductive environment within the joint, treadmill exercise provided a more satisfactory morphological outcome regarding the number of chondrocytes in the histological evaluation. As an outcome, better results were found in groups submitted to exercise, mostly treadmill exercise.

## 1 Introduction

Osteoarthritis (OA) is considered an inflammatory and degenerative joint disease, characterized by loss of hyaline joint cartilage and adjacent bone remodeling with the formation of osteophytes, accompanied by various degrees of functional limitation and reduction in the quality of life of individuals (Green et al., 2018; Rios et al., 2018). Given its avascular nature, cartilage is a tissue of difficult repair, which constitutes a major therapeutic challenge due to the high incidence in the world population (Shikichi et al., 1999; IWANAGA et al., 2000).

Recent studies on the pathophysiology of the disease indicate that the initiation and progression of OA are directly linked to the occurrence of an inflammatory process, cartilage fragmentation, and a state of oxidative stress in the joint environment (Henrotin et al., 2005; Okin and Medzhitov, 2012; Li et al., 2013; Zahan et al., 2020).

Removal of the meniscus encourages instabilities and overload on the point of load distribution in the cartilage, stimulating mechanical stress, inflammatory processes, and oxidative stress (Serra and Soler, 2019; Filho et al., 2021). In this way, the model works to promote strong OA triggering factors, similar to the involvement in human disease (Logerstedt et al., 2010; McCoy, 2015; Filho et al., 2021).

Regular physical exercise is well established in the literature as an important therapeutic ally in the prevention or treatment of several chronic diseases (RADak et al., 1999; Petersen and Pedersen, 2005; Cifuentes et al., 2010; Green et al., 2018; Rios et al., 2018), including OA. The moderate intensity mechanical stress provided by exercise seems to positively modulate signaling pathways of the main inflammatory mediators involved in the pathophysiology of OA (IL1-b, TNF-a, IL6, and IFN-γ), which seems to favor the regulation of the synthesis of proteoglycans and collagen, attenuating the process of joint wear (Pinho et al., 2010; Finsterer, 2012; Lindsay et al., 2015). In addition, exercise generates an anabolic/protective response through increased expression of anti-inflammatory cytokines (IL10 and IL4) and growth factors (TGF-b) (Sellam and Berenbaum, 2010; Musumeci, 2016; Rios et al., 2018) and activation of the antioxidant defense system (SOD and GSH), generating attenuation in the production of reactive oxygen species (ROS) by chondrocytes (Kühn et al., 2003; Henrotin et al., 2005; Altindag et al., 2007; Altay et al., 2015; Zahan et al., 2020).

Although the beneficial effects of physical exercises in this pathology are well established in the literature, there is still no agreement on which type of exercise brings more benefits in the treatment and management of OA (Batterham et al., 2011; Al-Hashem et al., 2017; Assis et al., 2018; Chen et al., 2020; Kolasinski et al., 2020; Tian et al., 2021). Therefore, from a literature review, it was identified that the most studied exercise protocols in animal models use treadmill protocols (Cifuentes et al., 2010; Moriyama et al., 2012; Assis et al., 2015; Assis et al., 2016; Assis et al., 2018) and swimming (Cechella et al., 2014; Assis et al., 2016; Tomazoni et al., 2016; Tomazoni et al., 2017; Assis et al., 2018; Hsieh and Yang, 2018).

Thus, this study aimed to investigate and compare the effects of two types of physical exercises widely used in the literature (treadmill vs. swimming) in a mechanical model of osteoarthritis to better understand the effects of these different modalities on the inflammatory response, oxidative stress markers and morphological variables present in OA.

## 2 Materials and methods

All experimental procedures involving animals were performed in accordance with the Guide for the Care and Use of Laboratory Animals of the National Institutes of Health (Bethesda, MD, United States) and with the approval of the Ethics Committee of the university (Universidade do Extremo Sul Catarinense—UNESC) with protocol number 51/2020. All animal experiments comply with the ARRIVE guidelines (Percie du Sert et al., 2020).

### 2.1 Animals

Forty-eight male Wistar animals (2 months old, 250–300 g) were kept at a controlled temperature of 20 ± 2°C, with a 12/12 h light/dark cycle and free access to food and water. The animals were randomly assigned to four experimental groups (*n* = 12 per group) as follows: Sham (without OA model induction), Osteoarthritis (OA); OA + Treadmill (T); OA + Swimming (S).

The number of animals was based on a review of studies with animal models (Galois et al., 2004; Moriyama et al., 2012; Assis et al., 2015; McCoy, 2015; Assis et al., 2016; Tomazoni et al., 2016; Al-Hashem et al., 2017; Tomazoni et al., 2017; Assis et al., 2018; Castrogiovanni et al., 2019; Serra and Soler, 2019; Chen et al., 2020; Percie du Sert et al., 2020; Tian et al., 2021), for the possibility of a difference of up to 20%–25% in the parameters to be analyzed between the groups, with a variance of up to 10% of the means, calculated using the EDA tool (du Sert et al., 2017), resulting in a sample size of 12 animals per group for biochemical and histological evaluations (7 animals per group for biochemical tests and 5 animals per group for histology analyses).

### 2.2 Osteoarthritis model

Rats were anesthetized with 4% isoflurane. The right knee was shaved, aseptically prepared with 90% alcohol, and exposed for surgery. For all groups, the same surgical approach was performed according to the standard incision performed in arthroplasty, prosthesis placement, and treatment of severe OA procedures in humans. This approach was also carried out in a previous experiment by this research group (Filho et al., 2021). It involves an anterior surgical approach to the knee, followed by medial parapatellar arthrotomy and lateral patellar dislocation, allowing access to the medial compartment of the knee of the animals (INSALL, 1971).

In OA groups, a meniscectomy of the medial meniscus was performed. Complete resection of the medial meniscus of the right hind limb was performed with a cold scalpel blade. In the Sham group, only the surgical approach was performed, without meniscectomy, followed by incision closure in two planes. There was no access to the lateral compartment of the joint and no additional ligament resection in any of the procedures. The central ligaments of the knee (anterior and posterior cruciate) and collateral ligaments (lateral and medial) were preserved. After reducing the patellar dislocation, the surgical incisions were closed in two planes with mono nylon sutures.

### 2.3 Intervention

Thirty days after the meniscectomy, the animals started the physical exercise protocols. The animals in the OA + T group were submitted to the prescription of treadmill exercise, according to a protocol adapted from Cifuentes et al. (2010), as can be seen in Table 1. The beginning took place with a week of adaptation (week 0), with each training session consisting of 10 min per day, on alternate days of the week, with a speed of 10 m/min, intending to adapt the animals to the protocol and the belt movement. During this week of adaptation, the animals received electrostimulation (0.2 mA), which served to stimulate the animal to walk and instruct it to move on the treadmill (Castrogiovanni et al., 2019).

**TABLE 1:** Moderate exercise protocol on treadmill.

Period	Velocity	Inclination (%)	Duration
Adaptation	10 m/min	1	10 min
Week 01	13 m/min	1	30 min
Week 02	13 m/min	1	30 min
Week 03	16 m/min	1	30 min
Week 04	16 m/min	1	50 min
Week 05	16 m/min	1	50 min
Week 06	16 m/min	1	50 min

The treadmill speed was 13 m/min, without incline. In weeks 01 and 02, the running time was 30 min; in weeks 03, 04, 05, and 06 the speed was 16 m/min. The execution time in these weeks was 30 min in week 03 and 50 min in weeks 04, 05, and 06. These training intensities and volumes correspond to light-moderate intensities, corresponding to approximately 50% and 60% of VO2max (Sutton et al., 2001; Cifuentes et al., 2010).

The animals in the OA + S group were submitted to the prescription of swimming exercise, in a specific tank designed for this type of study, with water at a temperature of 32°C, according to the protocol: the animals were submitted to an adaptation period of 20 min per day, during the first adaptation week (week 0), on alternate days. After the adaptation week, the animals started the swimming program, which developed on alternate days of the week, for 06 weeks. The protocol used was adapted from Cechella et al. (2014) and Hsieh and Yang (2018) and lasted 20 min per day, being carried out in alternating sessions during the weekdays, as can be seen in Table 2.

**TABLE 2:** Moderate swimming exercise protocol.

Period	Duration	Overload (%)
Adaptation	20 min	0
Week 01	20 min	3
Week 02	20 min	3
Week 03	20 min	3
Week 04	20 min	5
Week 05	20 min	5
Week 06	20 min	5

At the beginning of each training week, the animals were weighed and an overload, equivalent to the percentage of overload for the week, was attached to the tail of the animals with the aid of a sealed eppendorf containing lead. In weeks 01, 02, and 03, an overload equivalent to 3% of the body weight of each animal was used. For this, each rodent was weighed and its weight was recorded for weekly monitoring. In weeks 04, 05, and 06 an overload of 5% of body weight was placed on each animal.

The animals were considered unfit for training when they showed suffering like pain or discomfort that led them to: on the treadmill, not being able to follow the pace of the treadmill speed or dragging the paw (with OA) on the treadmill; in swimming, not being able to keep the head above the water surface or not performing swimming movements using the paw used for the OA model.

### 2.4 Euthanasia

**FIGURE 1:**
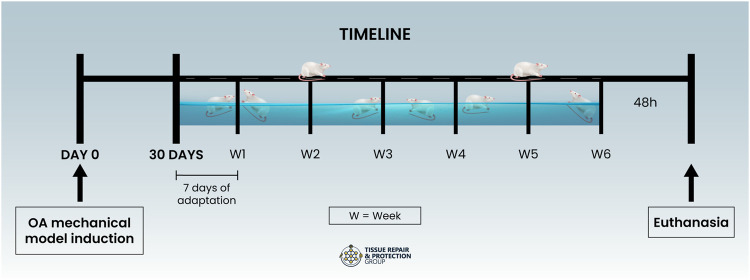
Timeline.

After these procedures, the animals were anesthetized with 4% isoflurane and killed by guillotine decapitation 48 h after the last training session (Figure 1), with the removal of gastrocnemius muscle samples to evaluate energy metabolism, a tissue sample from the joint in which all intra-capsular tissues of the joint were homogenized using a 7.4 pH sodium phosphate buffer (PBS) for biochemical analysis and distal femoral bony epiphysis with the cartilaginous surface, proximal tibial bony epiphysis with the cartilaginous surface, in addition to the lateral meniscus, for histological analyses.

### 2.5 Biochemical analysis

#### 2.5.1 Energy metabolism

Succinate Dehydrogenase Activity—Krebs Cycle: The activity of the enzyme succinate dehydrogenase was determined according to the method described by Fischer et al. (1985).

The activity of mitochondrial respiratory chain enzymes: Complex I activity was evaluated by the method described by Cassina and Radi (1996). Complex II activity was measured by the method described by Fischer et al. (1985).

#### 2.5.2 Intracellular determination of reactive oxygen species (ROS) and nitric oxide

The production of hydroperoxides was determined by the intracellular formation of 2′,7′-dichlorofluorescein (DCFHDA) from the oxidation of 2′,7′-dichlorodihydrofluorescein diacetate (H2DCFDA) by ROS (Dong et al., 2010).

The endothelial function was assessed by evaluating the nitric oxide levels by measuring its stable nitrite metabolite and quantified by spectrophotometer at 540 nm as described in the literature (Chae et al., 2004).

Both of the techniques were made with a standard curve in which the test resulted in high linearity of the samples, above 0.98, proving the high sensitivity.

#### 2.5.3 Determination of oxidative damage marker levels

The oxidative damage to protein was measured by the determination of carbonyl groups, based on a reaction with dinitrophenylhydrazine (DNTP), and the carbonyl contents were determined by measuring the absorbance at 370 nm (Levine et al., 1990).

Total thiol content was determined using the 5,5-dithiobis (2-nitrobenzoic acid) (2-nitrobenzoic acid) (DTNB) method, the absorbance at 412 nm was measured, and the amount of TNB formed (equivalent to the amount of sulfhydryl (SH) groups) was calculated (Aksenov and Markesbery, 2001).

#### 2.5.4 Determination of antioxidant defenses

SOD activity was quantified by inhibiting the oxidation of adrenaline and measured in a SpectraMax i3xELISA reader at 480 nm. Values were expressed as unit SOD/mg protein (U/mg protein) (Bannister and Calabrese, 1987).

Glutathione levels were measured through a reaction between DTNB and thiols, promoting color development as a result. Total glutathione (GSH) levels were expressed in µmol per mg of protein based on absorbance at 412 nm (Hissin and Hilf, 1976). This technique was made with a standard curve in which the test resulted in high linearity of the samples, above 0.98, proving the high sensitivity.

#### 2.5.5 Protein content

The protein content was determined using Folin phenol reagent (phosphomolybdic–phosphotungstic reagent) by Lowry et al. (1951). The bovine serum albumin was used to perform a standard curve. The results were expressed as mg protein (mg).

#### 2.5.6 Determination of the cytokine content using ELISA

The samples were processed and then the plate was sensitized for further incubation with the antibody. To measure cytokines (IFN-γ, TNF-α, IL1-β, IL6, IL4, IL10, and TGF-β) the enzyme-linked immunoabsorbent assay (Duoset ELISA) capture method (R&D system, Inc., Minneapolis, United States) was used.

### 2.6 Histological analysis

Samples extracted from the femoral condyle and tibial plateau regions of the right hind limb were soaked in 10% paraformaldehyde (PFA) solution in 0.1 M phosphate buffer (pH 7.4). Subsequently, they were fixed for 24 h in the same solution (PFA 10%), and embedded in paraffin after decalcification in 10% formic acid, dehydration, and bleaching, and sectioned into 5 µm thick sections. Histological quantifications of chondrocyte number, cartilage thickness, and cartilage-cartilage contact measure were performed by hematoxylin-eosin (H&E) staining (Moscardi et al., 2018). Slides were read under an optical microscope (Eclipse 50i, Nikon, Melville, NY, United States), at ×600 magnification, and four ocular fields were captured per slice (5 animals/group). Images were recorded using a Nikon camera (Sight DS-5M-L1, Melville, NY, United States) and analyzed using NIH ImageJ 1.36b software (NIH, Bethesda, MD, United States). The measurement of the chondrocyte number was determined in an area of 104 μm^2^ (Gonçalves et al., 2021). The measurement of cartilage-cartilage contact and cartilage thickness were both measured in the medial region of the joint.

To assess the degree of cartilage damage, samples were stained with Alcian Blue–Safranin O in five fields per slide per animal of each experimental group and analyzed according to the OARSI score described by Pritzker et al. (2006): 0–4 damage to cartilage, 5–6 additional damage to subchondral bones.

Histology analyzes were performed by a histopathologist who evaluated the number of chondrocytes count, measurement of cartilage thickness, measurement of cartilage-cartilage contact, and OARSI scale classification. These analyzes were performed blindly using numerical codes in the experimental groups.

### 2.7 Statistical analysis

Data are expressed as the mean ± standard error of the mean (SEM), evaluated by the Shapiro-Wilk normality test, and analyzed statistically by one-way analysis of variance (ANOVA) tests, followed by Tukey *post-hoc* test. The significance level for statistical tests is *p* < 0.05. GraphPad Prism version 7.0 (developed by GraphPad Software Inc.) was used as a statistical package.

## 3 Results

### 3.1 Energy metabolism

**FIGURE 2:**
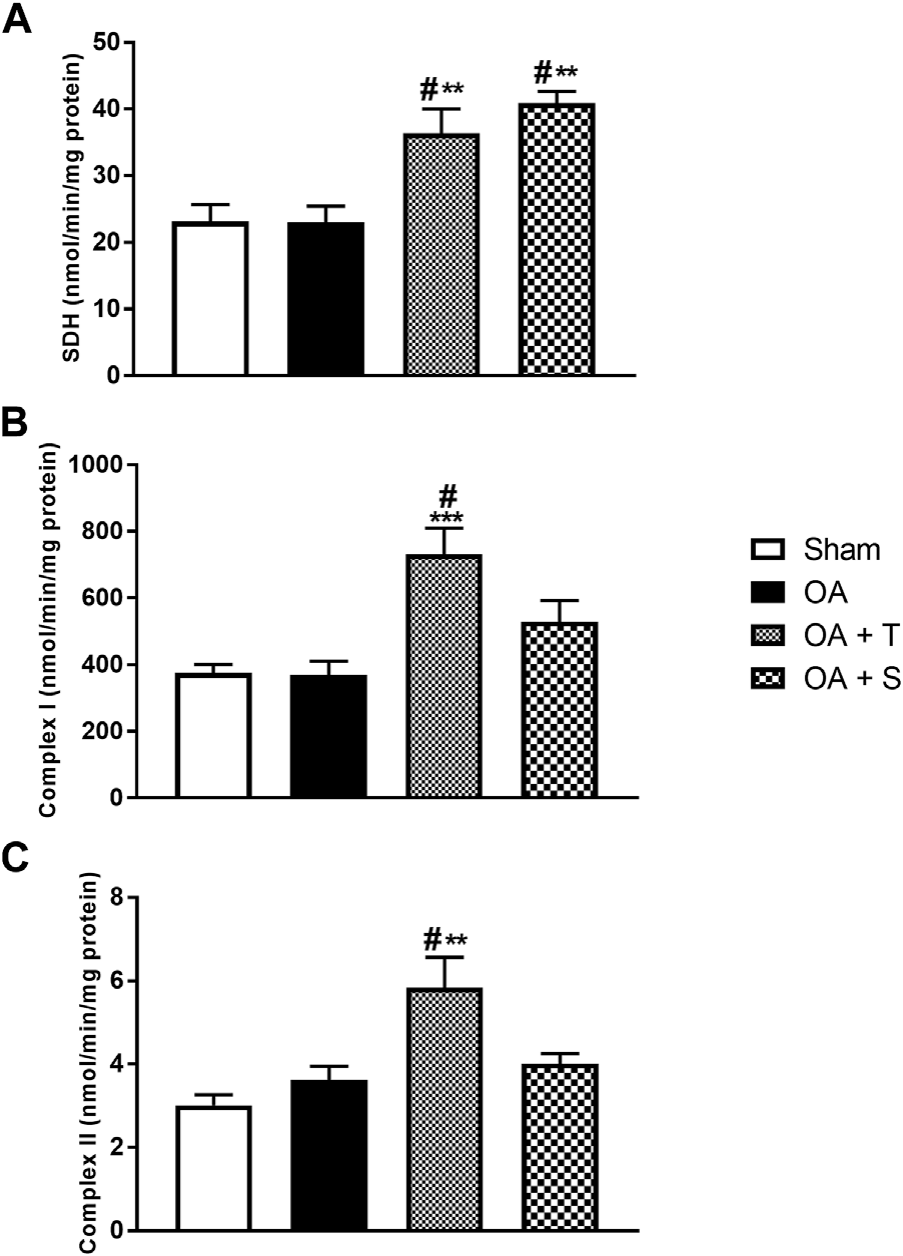
Activity of the components of the respiratory chain. **(A)** SDH; **(B)** Complex I; **(C)** Complex II. Abbreviations: OA, osteoarthritis; SDH, succinate dehydrogenase. Data are presented as mean ± SEM, in which: ^#^ *p* < 0.05 vs. Sham Group; **p < 0.01 vs. OA Group; ***p < 0.001 vs. OA Group; (One-way ANOVA followed by Tukey *post-hoc* test).

Figure 2 shows the levels of Succinate dehydrogenase (SDH) and complexes I and II of the electron transport chain (ETC). In Figure 2A, it is observed a significant increase in SDH in the OA + T and OA + S groups compared to the Sham group (*p* < 0.05). It is also possible to observe a statistical difference between the two, OA + T and OA + S, in relation to the OA group (*p* < 0.01). In Figure 2B it is observed a significant increase in the activity of complex I in the OA + T group compared to the Sham group (*p* < 0.05) and in relation to the OA group (*p* < 0.001). In Figure 2C, it is observed a significant increase in the activity of complex II in the OA + T group compared to the Sham group (*p* < 0.05) and in relation to the OA group (*p* < 0.01).

### 3.2 Oxidants levels

**FIGURE 3:**
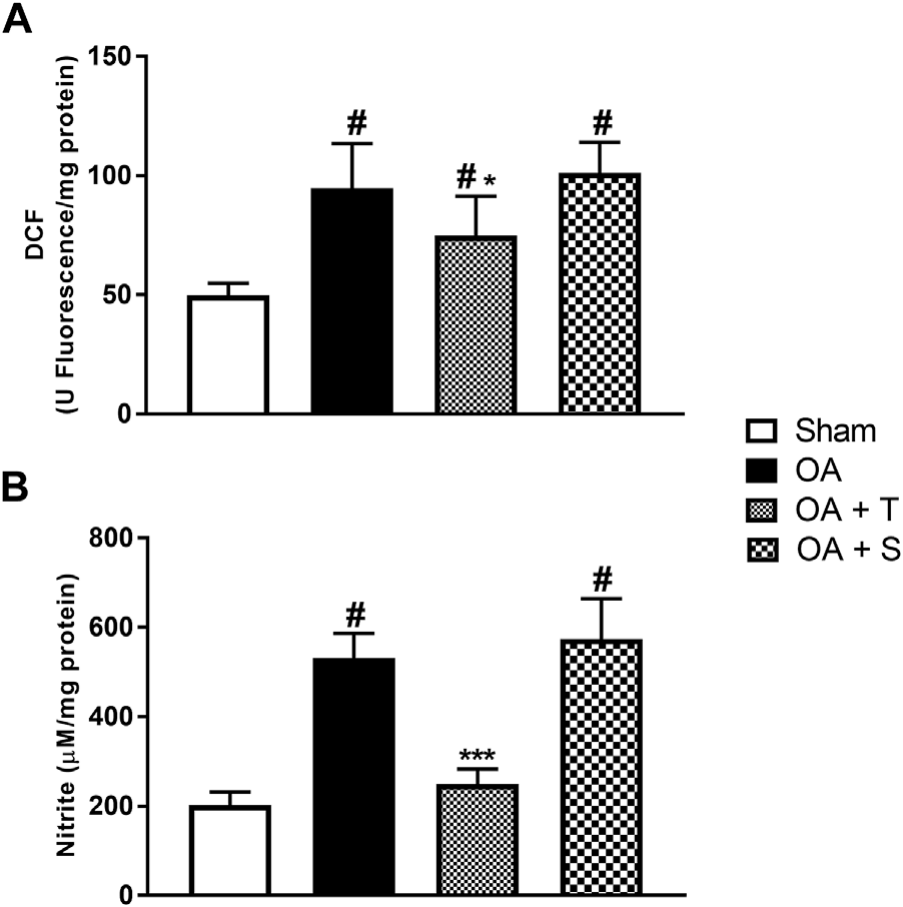
Oxidants. **(A)** DCF; **(B)** Nitrite. Abbreviations: DCF, dichlorofluorescein; OA, osteoarthritis. Data are presented as mean ± SEM, in which: ^#^ *p* < 0.05 vs. Sham Group; *p < 0.05 vs. OA Group; ***p < 0.001 vs. OA Group; (One-way ANOVA followed by Tukey *post-hoc* test).

In Figure 3A it is observed a significative elevation of DCF in OA, OA + T, and OA + S groups in relation to Sham, while OA + T showed a significant reduction when compared to the OA group (*p* < 0.05). In Figure 3B, nitrite levels showed significant increases in the OA and OA + S groups when compared to Sham, while the OA + T group showed a significant reduction in nitrite concentrations when compared to the OA group (*p* < 0.001).

### 3.3 Oxidative damage and antioxidants levels

**FIGURE 4:**
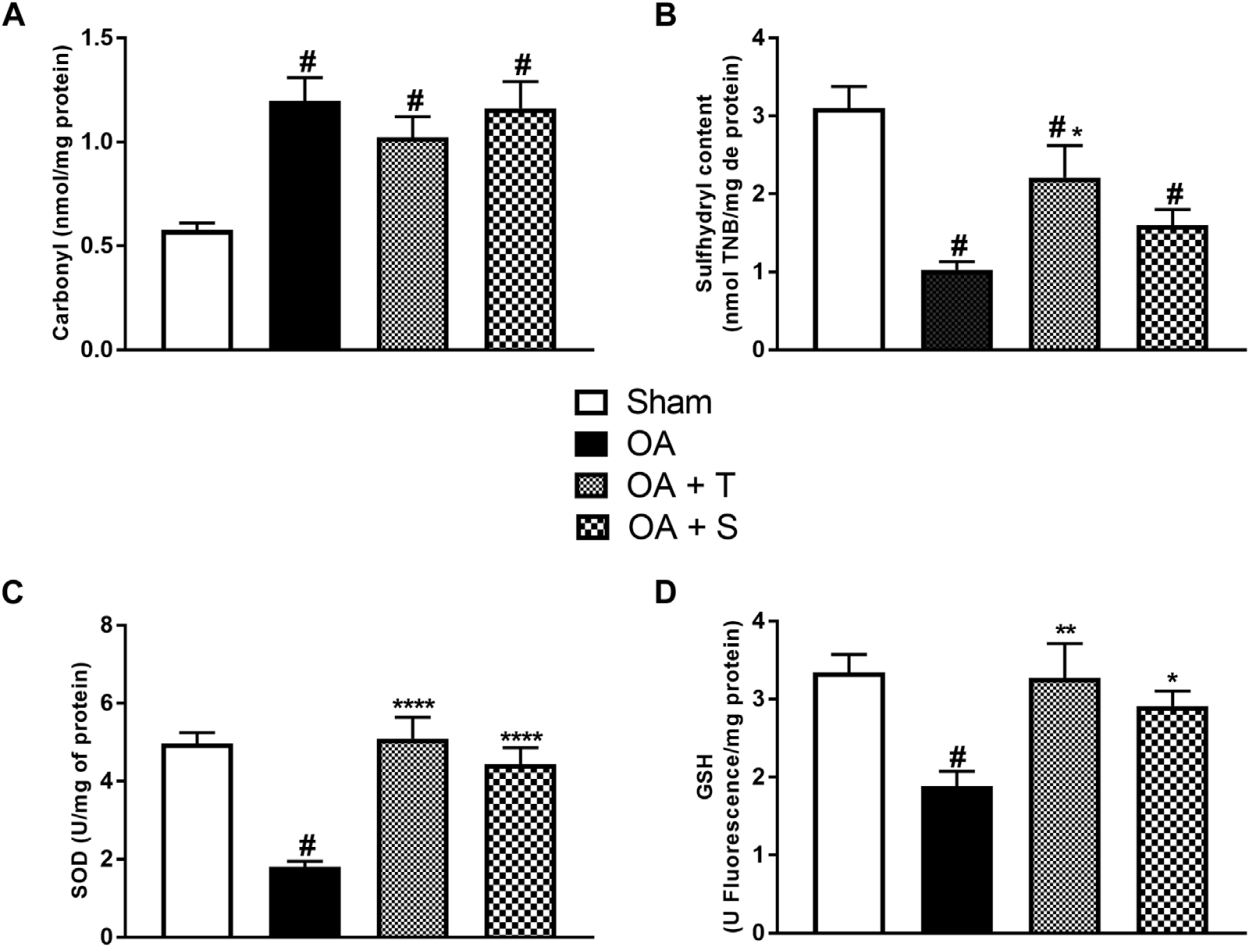
Oxidative damage and antioxidant system levels. **(A)** Carbonyl; **(B)** Sulfhydryl; **(C)**; SOD; **(D)** GSH. Abbreviations: GSH, reduced glutathione; SOD, superoxide dismutase. Data are presented as mean ± SEM, in which: ^#^ *p* < 0.05 vs. Sham Group; *p < 0.05 vs. OA Group; **p < 0.01 vs. OA Group; ****p < 0.0001 vs. OA Group; (One-way ANOVA followed by Tukey *post-hoc* test).

To assess oxidative damage, carbonyl levels and sulfhydryl content were analyzed (Figure 4). In 4A it is observed a significant increase of carbonyl was in the OA, OA + T, and OA + S groups in relation to the Sham group (*p* < 0.05). In 4B it is observed a significant reduction of sulfhydryl in the OA, OA + T, and OA + S groups in relation to the Sham group, while the OA + T group showed a significant increase when compared to the OA group (*p* < 0.05).

To assess the activity of the antioxidant system, SOD activity and GSH levels were measured. In 4C it is observed a significant reduction of SOD in the OA group in relation to the Sham group, while the OA + T and OA + S groups presented significant increases in SOD when compared to the OA group (*p* < 0.0001). In 4D it is observed a significant reduction of GSH in the OA group, in relation to the Sham group (*p* < 0.05). The OA + T group showed a significant increase (*p* < 0.01) when compared to the OA group. The OA + S group also showed significant increases in SOD when compared to the OA group (*p* < 0.05).

### 3.4 Pro-inflammatory cytokines

**FIGURE 5:**
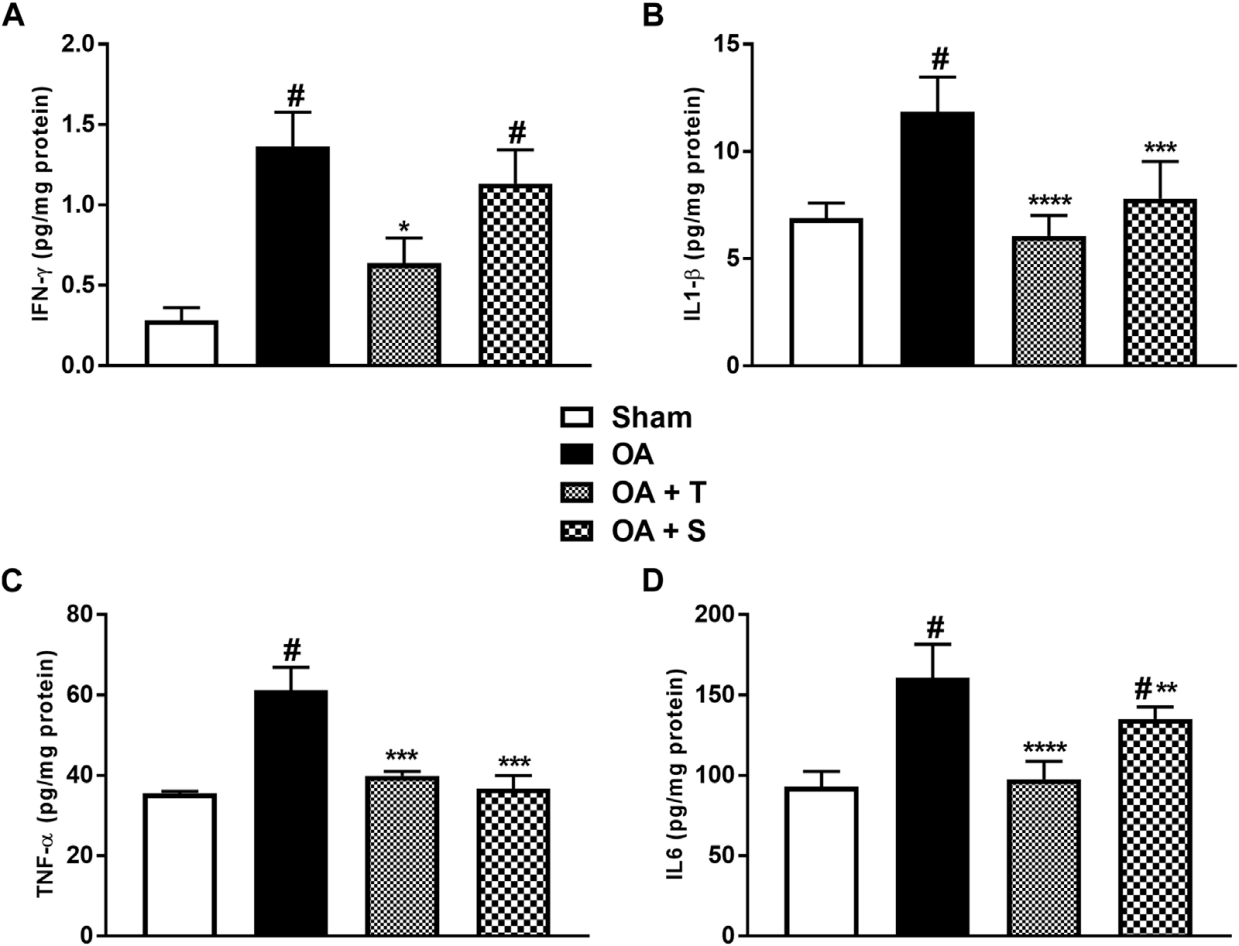
Pro-inflammatory cytokines. **(A)** IFN-ʎ; **(B)** IL1-β; **(C)** TNF-α; **(D)** IL6. Abbreviations: IL, interleukin; IFN, interferon; OA, osteoarthritis; TNF, tumor necrosis factor. Data are presented as mean ± SEM, in which: ^#^ *p* < 0.05 vs. Sham Group; *p < 0.05 vs. OA Group; **p < 0.01 vs. OA Group; ***p < 0.001 vs. OA Group; ****p < 0.0001 vs. OA Group; (One-way ANOVA followed by Tukey *post-hoc* test).

Figure 5 shows the levels of pro-inflammatory cytokines IFN-γ, IL1-β, TNF-α, and IL6. It is observed in Figure 5A that the OA and OA + S groups showed significant increases in IFN-γ in relation to the Sham group, while the OA + T group showed a significant decrease in relation to the OA group (*p* < 0.05). In 5B it is observed a significant increase of IL1-β in the OA group in relation to Sham (*p* < 0.05), while OA + T and OA + S groups showed significant reductions in the levels of IL1-β when compared to the OA group (*p* < 0.0001 e *p* < 0.001), respectively. In 5C, it is shown that TNF-α showed a significant increase in the OA group compared to the Sham group (*p* < 0.05), while the OA + T and OA + S groups showed significant reductions in this marker when compared to the OA group (*p* < 0.001). In 5D, we observed a significant increase in IL6 in the OA and OA + S group compared to the Sham group (*p* < 0.05). A decrease in IL6 concentration was observed in both types of physical exercise when compared to the OA group. The OA + S group presented (*p* < 0.01), while the OA + T group presented (*p* < 0.0001).

### 3.5 Anti-inflammatory cytokines

**FIGURE 6:**
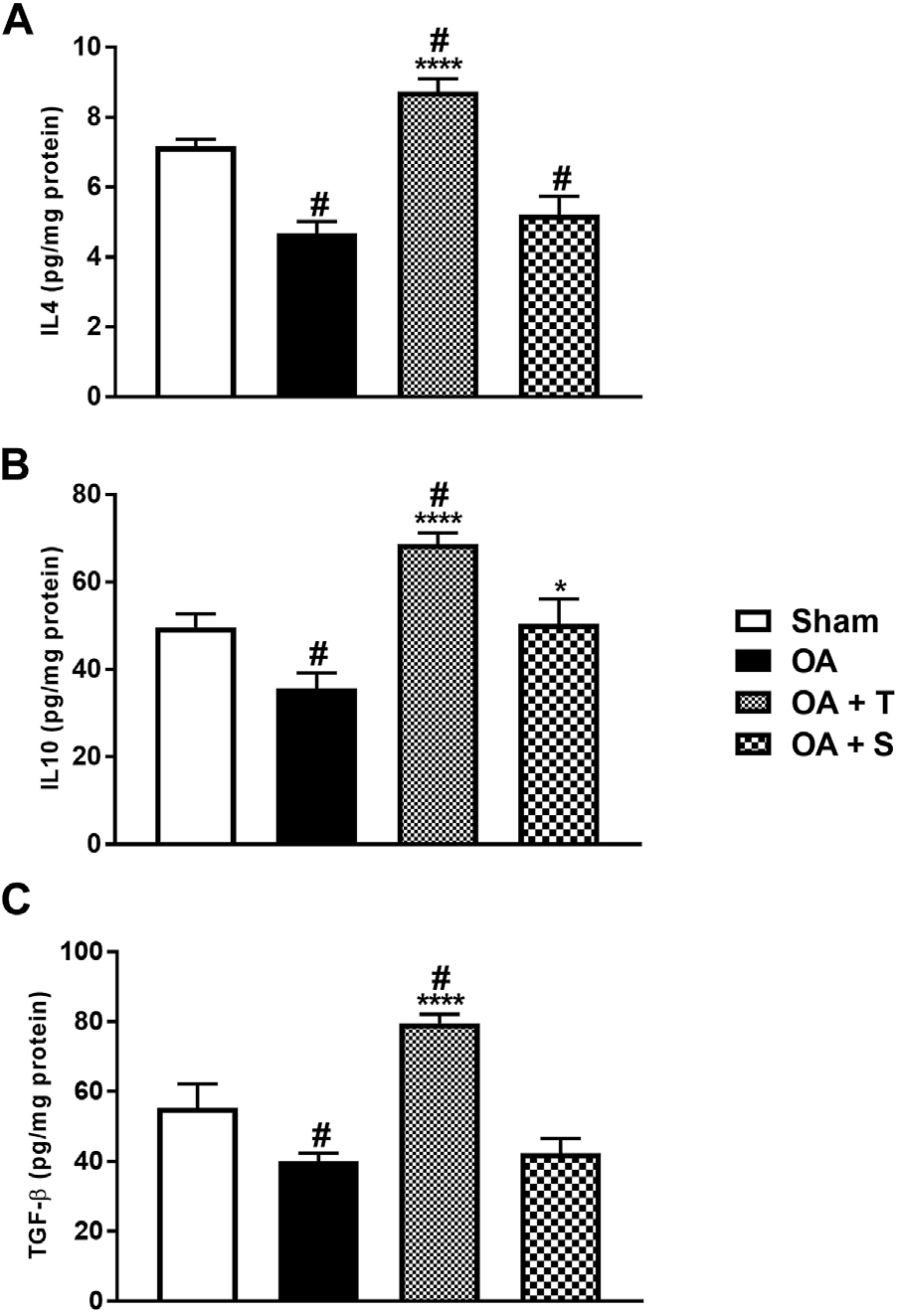
Anti-inflammatory cytokines. **(A)** IL4; **(B)** IL10; **(C)** TGF-β. Abbreviations: IL, interleukin; OA, osteoarthritis; TGF, transforming growth factor. Data are presented as mean ± SEM, in which: ^#^ *p* < 0.05 vs. Sham Group; *p < 0.05 vs. OA Group; ****p < 0.0001 vs. OA Group; (One-way ANOVA followed by Tukey *post-hoc* test).

Figure 6 shows the levels of anti-inflammatory cytokines IL4, IL10, and TGF-β. In 6A, it is observed a significant decrease of IL4 in the OA, OA + T, and OA + S groups in relation to Sham (*p* < 0.05), while the OA + T group showed a significant increase in IL4 levels when compared to the OA group (*p* < 0.0001). In 6B it is observed a significant decrease of IL10 in the OA group in relation to Sham (*p* < 0.05) and increase of IL10 in OA + T group in relation to OA group (*p* < 0.0001). In Figure 6C it is observed a significant decrease in TGF-β in the OA and OA + S groups compared to the Sham group, while the OA + T group showed a significant increase in TGF-β levels when compared to the OA group (*p* < 0.0001).

### 3.6 Histological analysis

**FIGURE 7:**
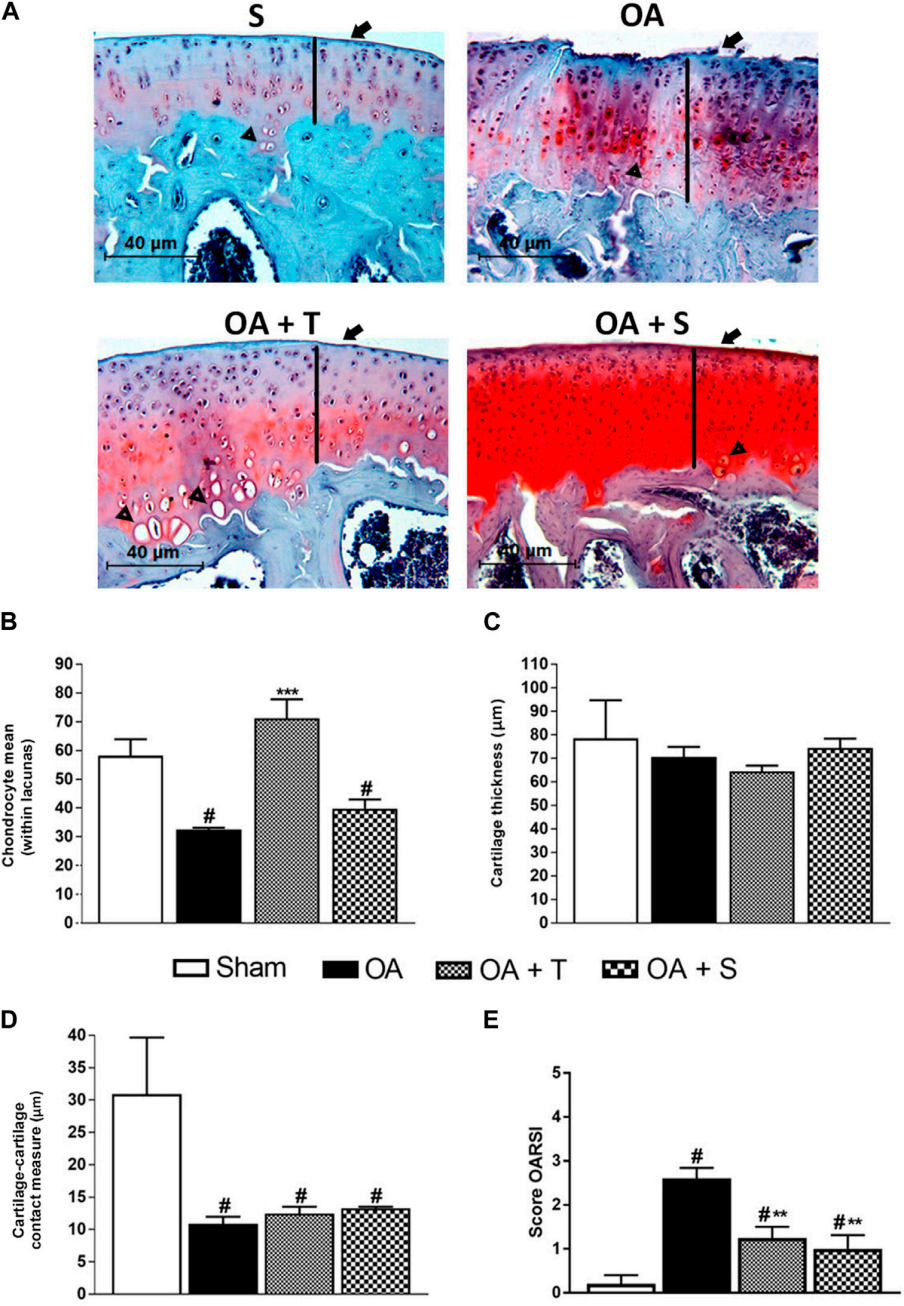
Histological analysis. **(A)** Representative images where Arrows = surface cartilage thickness; Arrowhead = chondrocytes inside the lacuna; Black line = cartilage thickness; **(B)** Chondrocyte mean; **(C)** Cartilage thickness; **(D)** Cartilage-cartilage contact; **(E)** Score OARSI. Data are presented as mean ± SEM, in which: ^#^ *p* < 0.05 v.s Sham Group; ***p < 0.001 v.s OA Group; (One-way ANOVA followed by Tukey post hoc test).

In Figure 7A, there are representative images of the histological analysis. In Figure 7B, we evaluated the mean number of chondrocytes inside the lacuna (40 µm). We observed that the OA and OA + S groups showed a significant decrease in the number of chondrocytes per lacuna when compared to the Sham group (*p* < 0.05). On the other hand, the OA + T group showed a significant increase in chondrocytes per lacuna when compared to OA group (*p* < 0.001). In Figure 7C, no significant change was observed between the groups in terms of cartilage thickness. In Figure 7D, in the cartilage-cartilage contact measure, the OA, OA + T, and OA + S groups showed statistically significant reductions when compared to Sham (*p* < 0.05).

In Figure 7E, the assessment of the degrees of cartilage injury according to the classification of the Osteoarthritis Research Society International (OARSI). In this evaluation, all groups submitted to the surgical model of OA had a higher degree of injury compared to the sham group (*p* < 0.05). However, the exercise intervention groups resulted in a lower degree of injury when compared to the OA group (*p* < 0.01).

Please see Supplementary Image 2 for a summary of all results.

## 4 Discussion

In the present study, moderate-intensity exercise interventions were used in a mechanical model of OA in Wistar rats. To analyze whether the protocols were able to generate physiological changes in the treated animals, succinate dehydrogenase (SDH), a metabolic marker of mitochondrial activity in the Krebs cycle and the electron transport chain (ETC) (Chilibeck et al., 1998) was evaluated. This marker showed high levels of activity in the two exercised groups compared to the control, showing that both physical exercise protocols provided changes in the aerobic pattern of the animals and suggesting that the exercise intervention was able to generate an increase in mitochondrial activity (Chilibeck et al., 1998; Kang et al., 2009; da Luz Scheffer and Latini, 2020) and greater activity in complexes I and II in ETC, that is, confirming that the physical exercise protocols produced metabolic alterations.

In addition, the proposed exercise was able to promote a reduction in inflammatory parameters and oxidative stress, generating less tissue damage, especially when performed on a treadmill. The swimming exercise, despite promoting movement resistance, causes less impact on the joint due to the presence of thrust generated by the water column (Cechella et al., 2014; Hsieh and Yang, 2018). On the other hand, treadmill exercise seems to enable adequate mechanical resistance and impact intensity, modulating anabolic signaling within the joint environment (Shikichi et al., 1999; IWANAGA et al., 2000; Henrotin et al., 2005; Green et al., 2018). The impact on the joint is related to OA triggering factors, in adequate proportions it stimulates the best perfusion of nutrients and oxygen between cartilage and synovial fluid (Castrogiovanni et al., 2019).

Corroborating the results of the present research, other sources also point out the decrease in the production of markers such as TNF-α and IL1β and the increase in cytokines such as IL10 and IL4 as an effect of moderate exercise, reinforcing the anti-inflammatory nature of the modality (Pinho et al., 2010; Tomazoni et al., 2017; da Luz Scheffer and Latini, 2020; Cerqueira et al., 2020; Baker et al., 2011; Woodell May and Sommerfeld, 2020; Gleeson et al., 2011).

In this study, it was shown that both moderate exercises on a treadmill and swimming were able to modulate the pro-inflammatory process from the reduction of cytokines such as TNF-α, IL1-β, and IL6. Furthermore, in the specific case of treadmill exercise, there was a decrease in IFN-γ. Such circumstances point to a possible faster phenotypic switch from M1 to M2, attenuating the acute inflammatory phase and, together, providing an “anti-catabolic” environment within the joint (Gleeson et al., 2011).

Furthermore, the reduction of damage caused by sustained inflammation to articular cartilage may also be associated with the pleiotropic action of IL6. Despite being considered an originally pro-inflammatory cytokine, research indicates that, during the training action, myocytes produce IL6 with an anti-inflammatory profile controlled by the action of CA2+ and glycogen-activated protein kinase (MAPK) (da Luz Scheffer and Latini, 2020; Gleeson et al., 2011; Benatti and Pedersen, 2015). This IL6 can induce negative feedback that inhibits the production of TNF-α by type “A” joint synovitis cells. In addition, it also promotes the production of the IL1-β antagonist receptor, the cytokine called IL1-RA, and the anti-inflammatory interleukin IL10, contributing to the minimization of the acute inflammatory process (Gleeson et al., 2011; Benatti and Pedersen, 2015; Castrogiovanni et al., 2019).

In the study, a reduction of IL6 was found in the samples of the groups treated with exercise, mainly in the exercise groups performed on the treadmill. This may be because IL6 with an anti-inflammatory profile peaks during muscle contraction during exercise, followed by an important gradual decrease soon after the end of the activity (Fischer, 2006; Benatti and Pedersen, 2015). Thus, as the material for analysis of this cytokine was removed 48 h after the last exercise session, it is understood that the evaluated IL6 has a pro-inflammatory profile probably associated with the characteristics of osteoarthritic disease.

However, it is estimated that during the training of the treated groups, IL6 at times of muscle contraction behaved as an anti-inflammatory cytokine that corroborated the increase in the production of anti-inflammatory cytokines, the inhibition of TNF-α and restriction of the action of IL1-β, mainly due to the chronic adaptive effect after sequential acute sessions of moderate-intensity exercise (Silva et al., 2018).

Furthermore, the results presented here confirm the increase in the concentrations of anti-inflammatory cytokines, among them IL10 and IL4, acting with a chondroprotective action in the trained groups.

IL10 is involved in the decrease of the expression of MMPs and the decrease in the synthesis of IL1-β and TNF-α (Molnar et al., 2021). It is synthesized by immune cells and chondrocytes, playing a prominent role in the physiological maintenance of ECM cartilage, as it has chondroprotective properties through stimulation of type II collagen synthesis (Mostafa Mtairag et al., 2001; Schulze-Tanzil et al., 2009; Wojdasiewicz et al., 2014). Literature data indicate that moderate exercise therapy can positively modulate IL10 synthesis, thus blocking joint damage (Fernandes et al., 2002; Mathiessen and Conaghan, 2017). Another mechanism involved in the intra-articular production of IL10 refers to the phenotypic change from M1 to M2 macrophages, stimulated by physical training. This change from M1 to M2 allows the synthesis of IL10, providing the emergence of a chondroprotective anabolic environment, triggered by the cartilage exposure to appropriate tensions, together with a positive outcome of anti-inflammatory effects from the prescription of moderate exercise (Mostafa Mtairag et al., 2001; Fernandes et al., 2002; Mathiessen and Conaghan, 2017).

As for IL4, its signaling pathway is not yet fully understood, but it is speculated that IL4 production is associated with Th2 cells that infiltrate the synovial membrane during the OA process (Ishii et al., 2002). It has been described to play an important role in joint chondroprotection, also inhibiting the secretion of MMPs, thus ultimately minimizing the degradation of proteoglycans, observed in the natural course of untreated OA (van Meegeren et al., 2012).

In addition to the presence of chronic inflammatory conditions, capable of generating morphological changes in the joint structure, the etiopathogenesis and progression of OA seem to be directly related to the cellular redox balance of the articular cartilage components (Bondeson et al., 2010; Woodell May and Sommerfeld, 2020).

The intensity of exercise execution is a relevant factor in the production of ROS (Zahan et al., 2020; Cifuentes et al., 2010; da Luz Scheffer and Latini, 2020). When performed at moderate intensity (Sutton et al., 2001; Leandro et al., 2007; Cifuentes et al., 2010), physical exercise can modulate the oxidative stress parameters either by increasing the activity of antioxidant enzymes, or by decreasing the production of oxidants, or even by lower the impairment of oxidized proteins (Leeuwenburgh and Heinecke, 2001; Filho et al., 2021), as observed in the groups submitted to exercise.

The benefits generated by exercise practice against oxidative stress markers can be explained by the mechanism of the adequacy of the antioxidant defense enzymatic system (SOD and GPX) and by the increase in tissue resistance to oxidative damage developed by physical exercise.

In the present study, physical exercise also caused a reduction in the levels of Dichlorofluorescein (DCF), an indirect marker of hydrogen peroxide (H2O2), which, within the chondrocytes, is capable of suspending the synthesis of proteoglycans and altering the synthesis of ATP in the CTE (Johnson et al., 2000; Migita et al., 2001), damaging the articular cartilage. In addition to the benefits mentioned above, physical exercise increases the activity of proteasomes, considered protein complexes directly related to the protein repair process against OS present in the osteoarthritic joint (RADak et al., 1999; da Luz Scheffer and Latini, 2020).

The proposed intervention protocols provided biochemical and molecular changes in the treated groups, modulating the inflammatory process and the formation of oxidative stress, through the stimulus imposed by moderate exercise in osteoarthritic joints. As a consequence of this process of improvement of inflammatory conditions and oxidative stress, the beginning of tissue repair is expected, which aims to reconstitute the injured tissues during the OA process.

In this therapeutic evolution of joint tissue repair, the chondrocyte plays a key role, being involved both in catabolic processes, becoming a source of MMP production, and in anabolic processes such as involvement in the synthesis of collagen II, proteoglycans, and growth factors within of the joint environment.

In parallel, growth factors such as TGF-β can stimulate chondrocytes to express cartilage-specific ECM molecules such as type II collagen and proteoglycans, promoting a tissue repair cycle (Silva et al., 2009; da Silva et al., 2009). This growth factor is usually associated with chondrogenesis, chondrocyte proliferation, accumulation of ECM components, and terminal differentiation (Van Der Kraan, 2018). Thus, from this interaction, it can be interpreted that the greater number of chondrocytes and levels of TGF-β found in the results of the study may be the result of this relationship to which treadmill exercise was able to modulate and which possibly had an influence of the anabolic states from the previously described anti-inflammatory and antioxidant actions.

In addition, experimental studies have shown that moderate physical exercise modulates IL1-β expression and increases the number of chondrocytes in histological sections of animals that underwent moderate physical training (Akkiraju and Nohe, 2015; Martins et al., 2019). Reinforcing this characteristic, research has revealed, through bioinformatics, that moderate-intensity exercise generates lower gene expression of caspase-3 and NF-kB in the joint. The first is related to cellular apoptosis and the second is associated with the secretion of pro-inflammatory cytokines such as IL1-β, IL6, and TNF-α (Galois et al., 2004; Qian et al., 2014; Yang et al., 2019; Lu et al., 2021). That is, the findings of the present study are in line with the literature and reaffirm this scenario, while an increase in the number of chondrocytes was observed compared to the OA group, especially in the group of physical exercise on the treadmill. It is estimated that this circumstance possibly stems from the better environment of cellular homeostasis provided by the mechanical action of treadmill physical exercise which, when appropriate, provides better delivery of nutrients and oxygen to the cartilage.

On the other hand, it was confirmed that OA plays pathophysiological mechanisms involved in the apoptosis of chondrocytes, since, when analyzing the quantity by a lacuna of these cells, a significant decrease is found in the OA group in relation to the Sham group.

Although no significant histological changes were found in the total thickness of cartilage and cartilage-cartilage contact measure between the exercised groups and the OA group, the degrees of injury in the OARSI score and the analysis of biochemical parameters suggest that the osteoarthritis group may have with the progressive degradation of cartilage, characteristic of the disease. On the other hand, exercise, especially when performed on a treadmill, seems to exert a protective factor on articular cartilage. Thus, it is considered a resource capable of preventing the progression and/or changing the speed and severity of tissue damage due to the control of factors associated with the etiology and progression of the disease, such as inflammatory parameters and oxidative stress, in addition to the benefit obtained by the practice, expressed by the increase in the number of chondrocytes and the lower degree of injury when compared to the OA group.

However, the treatment of OA is, therefore, a major therapeutic challenge due to the avascular nature and low cellularity of the cartilaginous tissue, which makes the tissue repair process difficult. Physical exercise, when compared with other treatment methods, such as surgical and pharmacological procedures, should be considered an effective form of management in the treatment and control of disease progression. In addition, it has the advantage of being a non-invasive approach, which, when properly prescribed, does not present side effects and generates systemic benefits, not restricted to the affected joint tissue.

Despite the biochemical and histological analyzes demonstrating protective effects on joint cartilage obtained by the protocols used, new studies containing the evaluation of cell signaling pathways may be useful for a more comprehensive understanding of the mechanisms of action of exercise on OA, based on the evaluation of aggrecanases and collagenases involved in the degenerative process, analysis of gene expression associated with anabolic and catabolic aspects of OA such as type II collagen and metalloproteinases, which may be considered limitations of the present study.

## 5 Conclusion

Moderate physical exercise was able to positively modulate inflammatory states, and cellular redox states, and, in the case of exercise on treadmill, it provided an increase in the number of chondrocytes. Thus, moderate exercise, especially on the treadmill, can be considered a convenient method for the treatment of osteoarthritis, capable of also providing benefits to numerous systems, not restricted only to the musculoskeletal system.

## Data Availability

The raw data supporting the conclusion of this article will be made available by the authors, without undue reservation.

## Supplementary material

Supplementary Material S1 ↑ increase; ↓ decrease; - no significant association; *p<0.05 v.s OA Group **p<0.01 v.s OA Group; ***p<0.001 v.s OA Group; ****p<0.0001 v.s OA Group.
