# Supplementary figures and images for: The effectiveness of treadmill and swimming exercise in an animal model of osteoarthritis
Source: Front Physiol. 2023 Feb 21;14:1101159. doi: 10.3389/fphys.2023.1101159 (PMC9990173; doi:10.3389/fphys.2023.1101159)

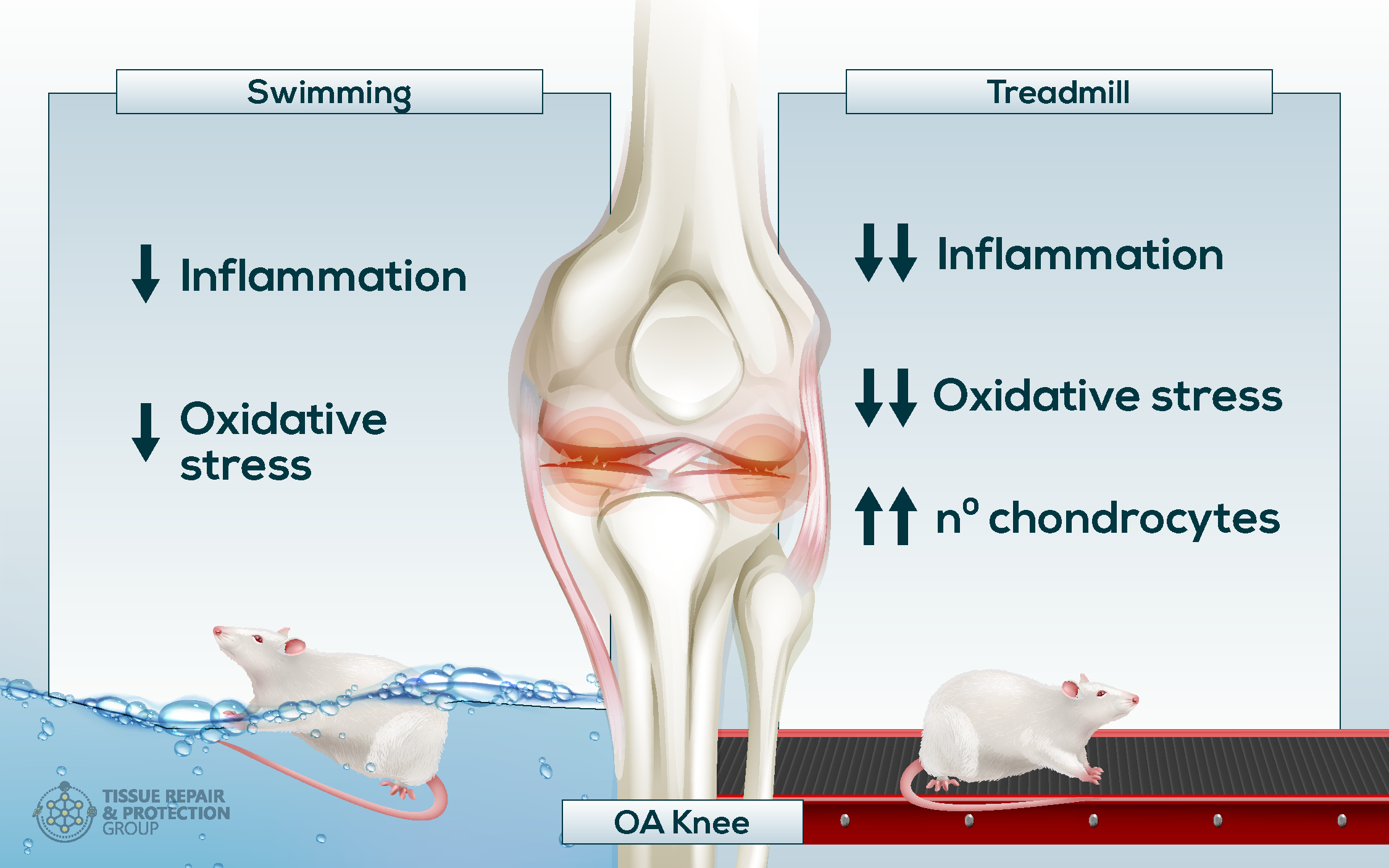

Supplement: Supplementary file 1 [file Image1.TIF]

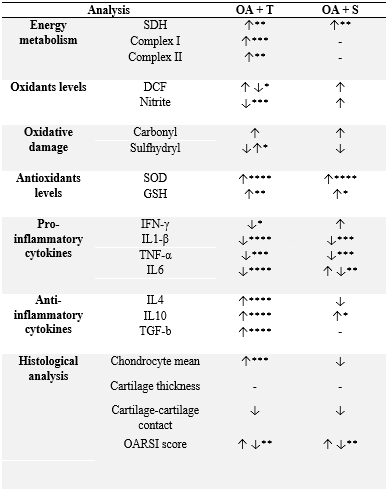

Supplement: Supplementary file 2 [file Image2.png]
